# All-trans retinoic acid promotes neural lineage entry by pluripotent embryonic stem cells via multiple pathways

**DOI:** 10.1186/1471-2121-10-57

**Published:** 2009-07-30

**Authors:** Jianfeng Lu, Li Tan, Ping Li, Hui Gao, Bo Fang, Shoudong Ye, Zhe Geng, Ping Zheng, Houyan Song

**Affiliations:** 1 Department of Molecular Genetics, Shanghai Medical School, Fudan University, Shanghai, PR China; 2 Key Laboratory of Molecular Medicine, Ministry of Education, PR China; 3 State Key Laboratory of Medical Neurobiology, PR China

## Abstract

**Background:**

All-trans retinoic acid (RA) is one of the most important morphogens with pleiotropic actions. Its embryonic distribution correlates with neural differentiation in the developing central nervous system. To explore the precise effects of RA on neural differentiation of mouse embryonic stem cells (ESCs), we detected expression of RA nuclear receptors and RA-metabolizing enzymes in mouse ESCs and investigated the roles of RA in adherent monolayer culture.

**Results:**

Upon addition of RA, cell differentiation was directed rapidly and exclusively into the neural lineage. Conversely, pharmacological interference with RA signaling suppressed this neural differentiation. Inhibition of fibroblast growth factor (FGF) signaling did not suppress significantly neural differentiation in RA-treated cultures. Pharmacological interference with extracellular signal-regulated kinase (ERK) pathway or activation of Wnt pathway effectively blocked the RA-promoted neural specification. ERK phosphorylation was enhanced in RA-treated cultures at the early stage of differentiation.

**Conclusion:**

RA can promote neural lineage entry by ESCs in adherent monolayer culture systems. This effect depends on RA signaling and its crosstalk with the ERK and Wnt pathways.

## Background

Embryonic stem cells (ESCs) are self-renewable and show a pluripotent ability to differentiate into cellular derivatives of all three primary germ layers. They are considered to be a unique biological system for disclosing the mechanisms of pluripotency and lineage commitment, and a useful material for disease modeling, pharmacological screening, and cell therapy.

All-trans retinoic acid (RA), which is a metabolic product of vitamin A (retinol), is one of the most important morphogens but with pleiotropic actions - it induces development of several lineages [1]. RA is an established signaling molecule involved in neuronal patterning, neural differentiation and axon outgrowth [2]. It is of great importance to understand well the mechanism of action of RA on the development of the nervous system. RA is known to be capable of inducing ESCs to differentiate into neurons within embryoid bodies (EBs) *in vitro *[3-5]. However, the route by which RA influences neural commitment is still obscure. Furthermore, it is difficult to dissect and manipulate differentiation within EBs because they are multicellular agglomerations of extra-embryonic endoderm and definitive ectodermal, mesodermal and endodermal derivatives [6].

In this study, we used a monolayer culture system of neural induction [7] and treated the cultures with low concentration of RA (10^-8 ^M), which ranged between the physiological (10^-9 ^M) and pharmacological (10^-6 ^M) levels [8]. We found that among RA nuclear receptors - *retinoic acid receptors (RARs) *and *retinoid *× *receptors (RXRs)*, and RA-metabolizing enzymes (*CYP26a1 *and *Raldh2*), only the mRNAs of *RARα, RARγ, RXRβ, Cyp26a1 *and *Raldh2 *were detectable in mouse ESCs. Upon stimulation with RA, the nuclear receptors and RA-metabolizing enzymes showed dynamic changes in expression. In RA-treated cultures, the specific marker for ESCs, i.e., *Oct4*, was down-regulated more rapidly, and markers for neural cells were up-regulated sooner than in the other two control groups. We reported that RA-promoted neural differentiation was blocked by the RARα-selective antagonist Ro 41-5253, which indicates that RA signaling is essential for neural specification in this monolayer culture system, and RARα pathway may play a key role in this effect. Furthermore, our data demonstrated that the process of this enhanced neural differentiation involved crosstalk between RA and the extracellular signal-regulated kinase (ERK) and Wnt pathways.

## Results

### 1. RA causes earlier expression of Sox1 in ESCs

To assess whether RA could promote ESCs entry to the neural lineage in a monolayer culture system, 46C ESCs were transferred into a differentiation induction regime which comprised an adherent monolayer culture without any exogenous growth factors [9], or with RA or solvent (0.02% ethanol). It is believed that the first conditions are permissive for neural commitment driven by autocrine signaling [7]. During experiments, we found that high does of RA (10^-6 ^M) showed an obvious noxious effect on cell survival (see Additional file 1 - Figure S1). Therefore, we treated the cultures with low concentration of RA (10^-8 ^M), which was between the physiological (10^-9 ^M) and pharmacological (10^-6 ^M) levels [10].

**Figure 1:**
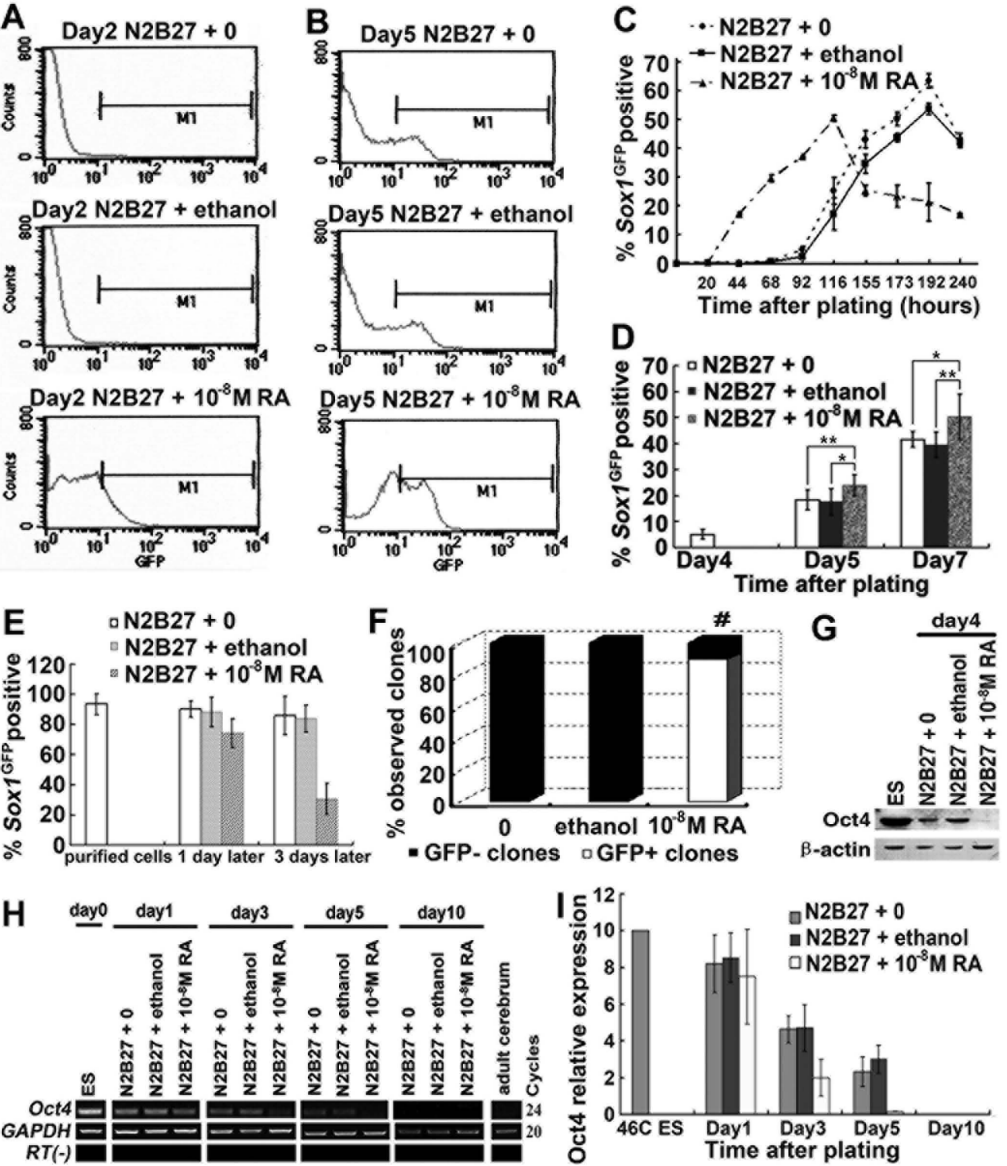
**RA stimulates accelerated neural specification**. 46C ESCs were cultured under monolayer differentiation conditions with RA (N2B27 + 10^-8 ^M RA) or without RA (N2B27 + 0 or N2B27 + ethanol). **A**, **B**, Typical FACS profile of *Sox1*^GFP ^expression at day 2. M1 is the gate used throughout to quantify the proportion *Sox1*^GFP^+ cells; **C-E**, Proportion of *Sox1*^GFP^+ cells at various time points (average of triplicates). *, p < 0.05; **, p < 0.01. **D**, Treatment of adherent cultures with RA after the appearance of the first *Sox1*^GFP^+ cells; **E**, Treatment of purified *Sox1*^GFP^+ cells with RA; **F**, Ratios of clones containing GFP+ cells (GFP+) or without GFP+ cells (GFP-) when cultured at clonal density. Under each culture condition, 28 clones were observed and counted. #, significant difference in this ratio from controls (p < 0.001, using contingency table analysis); **G**, Western blot analysis of Oct4 at day 4 under monolayer cultures; **H**, RT-PCR for the ESCs marker Oct4 during monolayer differentiation. To normalize their expression to the amount of cDNA present in the sample, the cDNA for endogenous GAPDH was amplified; **I**, Q-PCR for the ESCs marker Oct4 during monolayer differentiation.

Cells were harvested and analyzed by flow cytometry for *Sox1*^GFP ^expression, which is the earliest known specific marker of neuroectoderm in the mouse embryo [11]. 46C ESCs without treatment with any exogenous growth factors (N2B27+0, control) or after treatment with ethanol (N2B27+ethanol, solvent control) generated green fluorescent protein (GFP) positive cells progressively after an initial lag: almost no GFP+ cells were detected after 68 h, and only 4.6 ± 1.24% (N2B27+0) or 2.5 ± 0.58% (N2B27+ethanol) after 92 h. In contrast, 10^-8 ^M RA-treated cultures contained about 1% GFP+ cells at 20 h, which increased dramatically to 17.2 ± 0.30% at 44 h (Figure 1C).

10^-8 ^M RA increased the number of cells in the monolayer culture at the early stage of neural induction (see Additional file 2 - Figure S2). Therefore, it is reasonable to speculate that RA can also act after specification to increase the survival and/or proliferation of neural precursors. We cultured cells without RA for 96 h, and then treated the adherent cultures with RA. We observed an increase in the number of *Sox1*^GFP^+ cells after treatment with RA (Figure 1D). However, it is still hard to judge whether the increase in *Sox1*^GFP^+ cells was caused by the survival and/or proliferative effects of RA on neural precursors or by its neural differentiation effects on ESCs. Therefore, we obtained some purified *Sox1*^GFP^+ cells by culturing cells without RA for 6 days and selecting *Sox1*^GFP^+ cells by treatment with 0.5 μg/ml puromycin for another 4 days. These purified *Sox1*^GFP^+ cells were treated with or without RA for several days. After treatment with RA, there was a dramatic decrease in *Sox1*^GFP^+ cells (Figure 1E). Moreover, cultures at clonal density showed that there were almost no clones that contained GFP+ cells in control and solvent control cultures, while more than 80% of clones contained GFP+ cells in RA-treated cultures (Figure 1F). These data suggested that the observed initial increase in frequency of *Sox1*^GFP^-expressing cells was not caused mainly by the survival and/or proliferative effects of RA on neural precursors but by its neural differentiation effects on ESCs.

To test further whether RA directly promoted ESC differentiation, we examined the cell-density dependence of neural specification. Neural induction of ESCs during monolayer differentiation is strongly inhibited by even modest increases in cell density [9]. Cells treated with 10^-8 ^M RA generated far fewer *Sox1*^GFP^+ neural precursors at high cell density (3 × 10^4^cells/cm^2^) than those at low cell density (1 × 10^4^cells/cm^2^), while in control and solvent control cultures, cells almost completely eliminated neural specification at high density (see Additional file 3 - Figure S3). This suggested that RA affected neural specification of ESCs also in a cell-density dependent manner.

We also noted a difference in the variability of GFP intensity between cultures. Control or solvent control cultures contained a mixture of less intensely GFP+ cells [Figure 1B (Day5 N2B27+0 and Day5 N2B27+ethanol)], whereas RA-treated cells showed uniformly moderate GFP expression [Figure 1B (Day5 N2B27 + 10^-8 ^M RA)].

The effects of RA at the onset of ESC differentiation were indicated by the kinetics and pattern of gene regulation. One of the key marker genes *Oct4 *was examined. *Oct4*, a critical determinant of the pluripotent state, is down-regulated early during somatic differentiation [12]. Within 3 days of leukemia inhibitory factor (LIF) and serum withdrawal, *Oct4 *mRNA decreased dramatically in RA-treated cells, while there was a smaller decrease in control or solvent control cells; after day 3, almost no Oct4 expression was observed in RA-treated cultures (Figure 1G-I).

In summary, these data indicated that RA acted at the initial stages of ESC differentiation to promote the transition into neuroectoderm.

### 2. RA facilitates emergence of neural progenitors, neural stem cells and neurons

**Figure 2:**
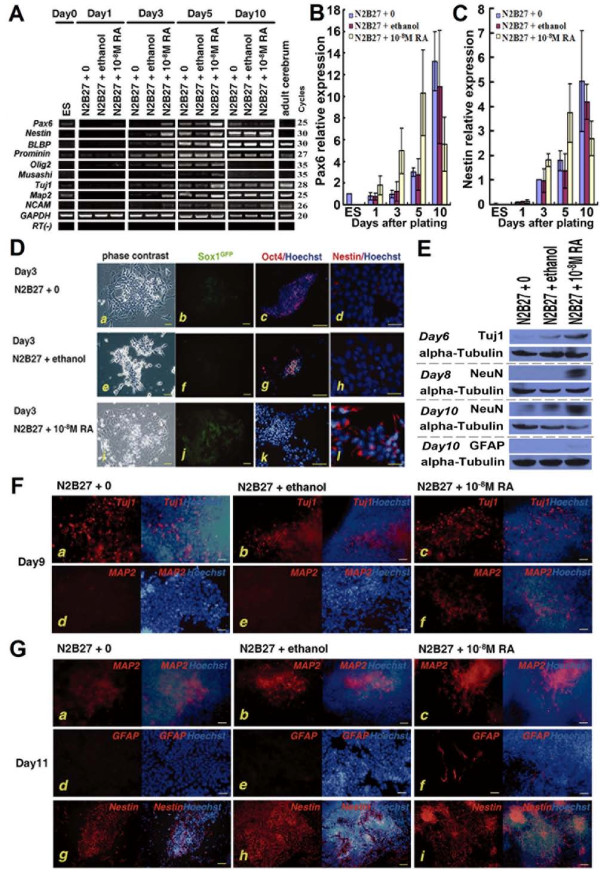
**RA facilitates emergence of neural cells**. ESCs were cultured under monolayer differentiation conditions with RA (N2B27 + 10^-8 ^M RA) or without RA (N2B27 + 0 or N2B27 + ethanol). **A**, Expression of neural markers during differentiation under monolayer cultures at different time points. RNA was isolated from ESCs (day 0), monolayer cultures (days 1, 3, 5 and 10) and adult mouse brain, and analyzed by RT-PCR for expression of markers of neural progenitors/stem cells and neurons. To normalize their expression to the amount of cDNA present in the sample, the cDNA for endogenous *GAPDH *was amplified; **B**, **C**, Q-PCR for the neural marker *Pax6 *and *Nestin *during monolayer differentiation; **D, F-G**, Cultures at different time points of monolayer differentiation, shown in phase contrast or stained for markers as indicated; **D**, Cultures at 72 h of monolayer differentiation showed that in RA-treated cultures, *Sox1*^GFP^+ and Nestin+ cells were found, while nearly no Oct4+ cells were detected; **E**, Western blot analysis of Tuj1 at day 6, of NeuN at day 8 and day 10, and of GFAP at day 10 under monolayer cultures; **F**, At day 9, though Tuj1+ cells were also found in control cultures, the marker of mature neurons MAP2 was only detected in RA-treated cultures; **G**, At day 11, neurons (MAP2+ cells) emerged in all cultures, however, the marker of glia cells GFAP was only found in RA-treated cultures with a very low percentage. In control cultures, cells were dominantly Nestin+, while in RA-treated cultures, the percentage of Nestin+ cells was much lower. Scale bars: D (a, b, e, f, i, j): 40 μm; D (c, g, k): 80 μm; D (d, h, l), F-G: 25 μm.

To ensure that RA indeed accelerated neural specification, we assayed multiple markers of neural progenitors, neural stem cells and neurons by RT-PCR and Q-PCR (Figure 2A-C). *Pax6 *(a marker for neuroectoderm) and *Nestin *(which is expressed in undifferentiated neural progenitors) were detected readily in RA-treated cultures at day 3, while no obvious expression was found in control and solvent control cultures. Other markers of neural stem/progenitor cells, such as *BLBP *(brain lipid binding protein) (a marker of more mature neural precursors), *Olig2 *(a basic-helix-loop-helix transcription factor that is expressed in most ventral neural progenitor cells around the period of neural tube closure [13]) and *Musashi *(which encodes an RNA-binding protein), were all expressed earlier in RA-treated cultures. *Tuj1 *(a marker of immature neurons), *NCAM *(neural cell adhesion molecule) (a nonexclusive neural marker) and *MAP2 *(microtubule associated protein 2) (a marker of mature neurons), were also expressed earlier in RA-treated cultures than in control or solvent control cultures.

We also used fluorescence microscopy and western blot to monitor the expression of markers for neural stem cells, neurons and glia. At day 3, with the rapid disappearance of Oct4+ cells, *Sox1*^GFP^+ and Nestin+ cells were found in RA-treated cultures (Figure 2D). The expression of Tuj1 was upregulated at day 6 in RA-treated cultures, while 3 days later, Tuj1+ cells appeared in control and solvent control cultures (Figure 2E and 2F). With the accelerated emergence of neural stem cells and immature neurons, the expression of Neuronal nuclei (NeuN; a vertebrate neuron-specific nuclear protein) and glial fibrillary acidic protein (GFAP; a marker of glia cells) was also upregulated, and MAP2+ and GFAP+ cells also appeared earlier in RA-treated cultures (Figure 2E-G). At day 11, in control and solvent control cultures, most cells were Nestin+ and were in the rosette conformations that is typical of neuroepithelial cells (Figure 2Gg and 2Gh), whereas in RA-treated cultures, although Nestin+ cells were still distributed in the rosette pattern, the percentage of Nestin+ cells was obviously lower (Figure 2Gi).

These findings indicated clearly that RA actually facilitated the differentiation of neural progenitors and neural stem cells, and as a consequence, neurons and glial cells appeared earlier in RA-treated cultures.

### 3. Requirement for the RA signaling during efficient neural specification of ESCs

**Figure 3:**
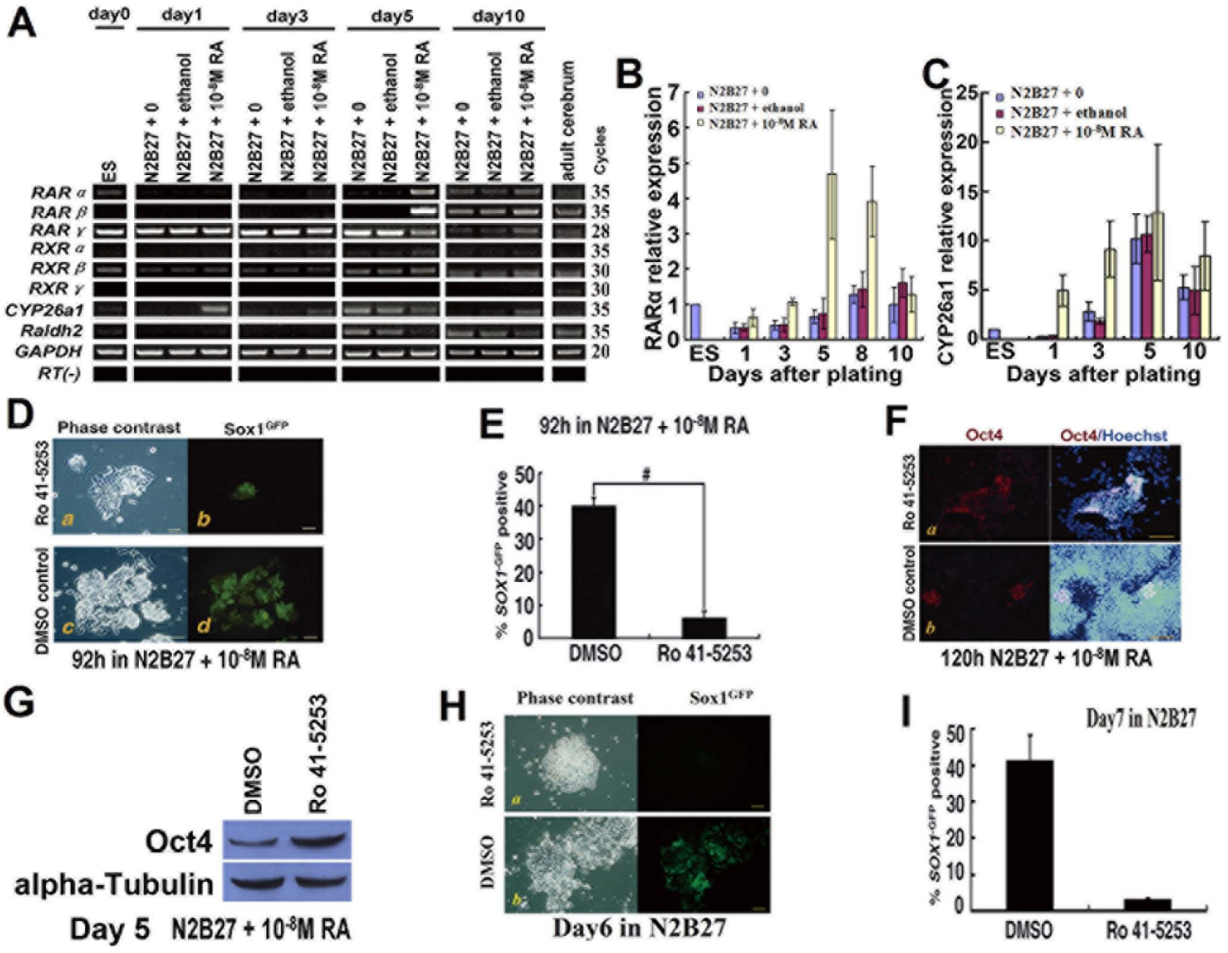
**Requirement for RA signaling for efficient neural specification of ESCs**. **A**, RT-PCR for RARs, RXRs and RA metabolism-relative enzymes. RNA was isolated from ESCs (day 0), monolayer cultures (days 1, 3, 5 and 10) and adult mouse brain. Expression of RA nuclear receptors and two RA-metabolizing enzymes (*CYP26a1 *and *Raldh2*) were analyzed by RT-PCR; **B**, **C**, Q-PCR for *RARα *and *CYP26a1 *during monolayer differentiation; **D-G**, Monolayer differentiation of 46C ESCs in RA-treated cultures (N2B27 + 10^-8 ^M RA) exposed to 2 μM RARα inhibitor Ro 41-5253 or to equivalent amounts of DMSO diluents; **D**, Cultures of monolayer differentiation, shown in phase contrast or stained for *Sox1*^GFP^; **E**, Percentage of *Sox1*^GFP^+ cells scored by FACS. #, significant difference between inhibitor-treated cultures and DMSO-treated cultures (p < 0.001); **F**, Cultures of monolayer differentiation, shown in stained for Oct4 or combination of Oct4 and Hoechst; **G**, Western blot analysis of Oct4 at day 5 under monolayer cultures; **H, I**, Monolayer differentiation of 46C ESCs in cultures without RA (N2B27) exposed to 2 μM RARα inhibitor Ro 41-5253 or to equivalent amounts of DMSO diluents at Day6; **H**, Cultures of monolayer differentiation, shown in phase contrast or stained for *Sox1*^GFP^; **I**, Percentage of *Sox1*^GFP^+ cells scored by FACS; Scale bars: D, 40 μm; F, H, 80 μm.

To assess the potential contribution of the RA pathway to ESC fate, we investigated the expression of RA nuclear receptors and RA-metabolizing enzymes in mouse ESCs. Among *RARs *(*RARα*, -*β *and -*γ*), *RXRs *(*RXRα*, -*β *and -*γ*) and RA-metabolizing enzymes (*Raldh2 *and *CYP26a1*), only *RARα, RARγ, RXRβ, Cyp26a1 *and *Raldh2 *were detected readily by RT-PCR of total RNA prepared from 46C ESCs (Figure 3A). Notably, at the early stage of differentiation, only the expression of *RARα*, *Raldh2 *and *CYP26a1 *showed obvious differences between RA-treated and control cultures (Figure 3A-C). This hinted that these three molecules might play important roles in the promoted neural specification effect of RA. Therefore, we used a pharmacological approach to interfere with the RARα pathway.

When 46C ESCs were exposed to the RARα-selective antagonist Ro 41-5253 [14] during the monolayer differentiation, the majority of cells failed to become *Sox1*^GFP^+ neural cells. After 92 h of differentiation, <10% of inhibitor-treated cells became *Sox1*^GFP^+ compared with around 40% of cells exposed to DMSO vehicle (Figure 3D and 3E, p < 0.001). Many inhibitor-treated cells remained Oct4+ during this period (Figure 3F and 3G). Ro 41-5253 also inhibited neural differentiation in the culture without RA (Figure 3H and 3I).

Our observations suggested that blockade of the RARα pathway initially impeded lineage commitment, and RA signaling was required for the efficient neural specification of ESCs.

### 4. RA acts upstream of MAP kinase/ERK kinase (MEK) to promote neural specification

**Figure 4:**
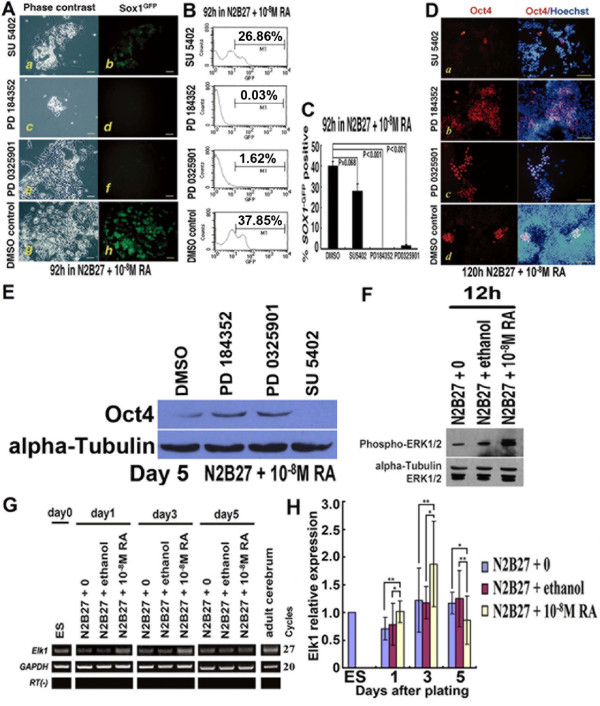
**RA could not bypass the requirement for ERK signaling in neural specification**. Monolayer differentiation of 46C ESCs in RA-treated cultures (N2B27 + 10^-8 ^M RA) exposed to FGF receptor tyrosine kinase inhibitor SU5402 (5 μM), MEK1/2 inhibitors PD184352 (4 μM) or PD0325901 (4 μM), or to equivalent amounts of DMSO diluents. **A**, Cultures of monolayer differentiation, shown in phase contrast or stained for *Sox1*^GFP^; **B**, Typical FACS profile of *Sox1*^GFP ^expression; **C**, Percentage of *Sox1*^GFP^+ cells scored by FACS; **D**, Cultures of monolayer differentiation, shown in stained for Oct4 or combination of Oct4 and Hoechst; **E**, Western blot analysis of Oct4 at day 5 under monolayer cultures; **F**, Western blot analysis of phosphorylated ERK1/2 and ERK1/2 at 12 h under monolayer cultures; **G**, RT-PCR for *Elk1*, one of the targets of ERK signaling; **H**, Q-PCR for *Elk1 *during monolayer differentiation. *, p < 0.05; **, p < 0.01. Scale bars: A, 40 μm; D, 80 μm.

In vertebrate embryos, neural induction requires fibroblast growth factor (FGF) signaling through the Ras-ERK pathway [15-17]. This also appears to be true for ESCs, in which the initial stimulus is most likely provided by autocrine FGF4 [9]. To test whether RA bypassed this requirement, we used three pharmacological inhibitors (Figure 4). SU5402 inhibits the FGF receptor tyrosine kinase [18]; PD184352 and PD0325901 are both able to block MEK1/2, upstream of ERK1/2 [19,20]. All of these inhibitors are able to block neural specification of ESCs [7,21,22]. RA-treated cells remained susceptible to blockade of neural induction by the two MEK1/2 inhibitors, PD184352 and PD0325901, while they were less susceptible to blockade of neural induction by FGF receptor tyrosine kinase inhibitor SU5402 (Figure 4A-C). Notably, SU5402 and PD184352 markedly decreased cell number in RA-treated monolayer cultures, while PD0325901 achieved the same suppressive effect on ERK activation as PD184352, without the dramatic side effect on cell viability or proliferation (see Additional file 4 - Figure S4). In the presence of SU5402, most cells lost the Oct4 marker of ESCs and differentiated under the driving force of RA. However, in the presence of the two MEK1/2 inhibitors, many cells continued to proliferate in an apparently undifferentiated Oct4+ state with minimal generation of *Sox1*^GFP^+ derivatives (Figure 4A-E).

To assess whether RA affected activation of the ERK pathway during neural specification, we detected phosphorylation of ERK by western blotting. Twelve hours after differentiation, a higher level of phosphorylated ERK was observed in RA-treated cultures (Figure 4F). The expression of the transcription factor *Elk1*, one of the downstream targets of ERK [23,24], was also up-regulated more in the RA-treated cultures than in the other two control cultures before day 3 (Figure 4G and 4H).

These findings indicated that RA acted upstream of MEK1/2 in the neural induction cascade. RA may play a role in enhancing ERK phosphorylation via MEK1/2 or its upstream but not in an FGF-signaling-dependent manner. The exact targets of RA remain to be determined.

### 5. Wnt signaling is involved in RA-promoted neural specification

**Figure 5:**
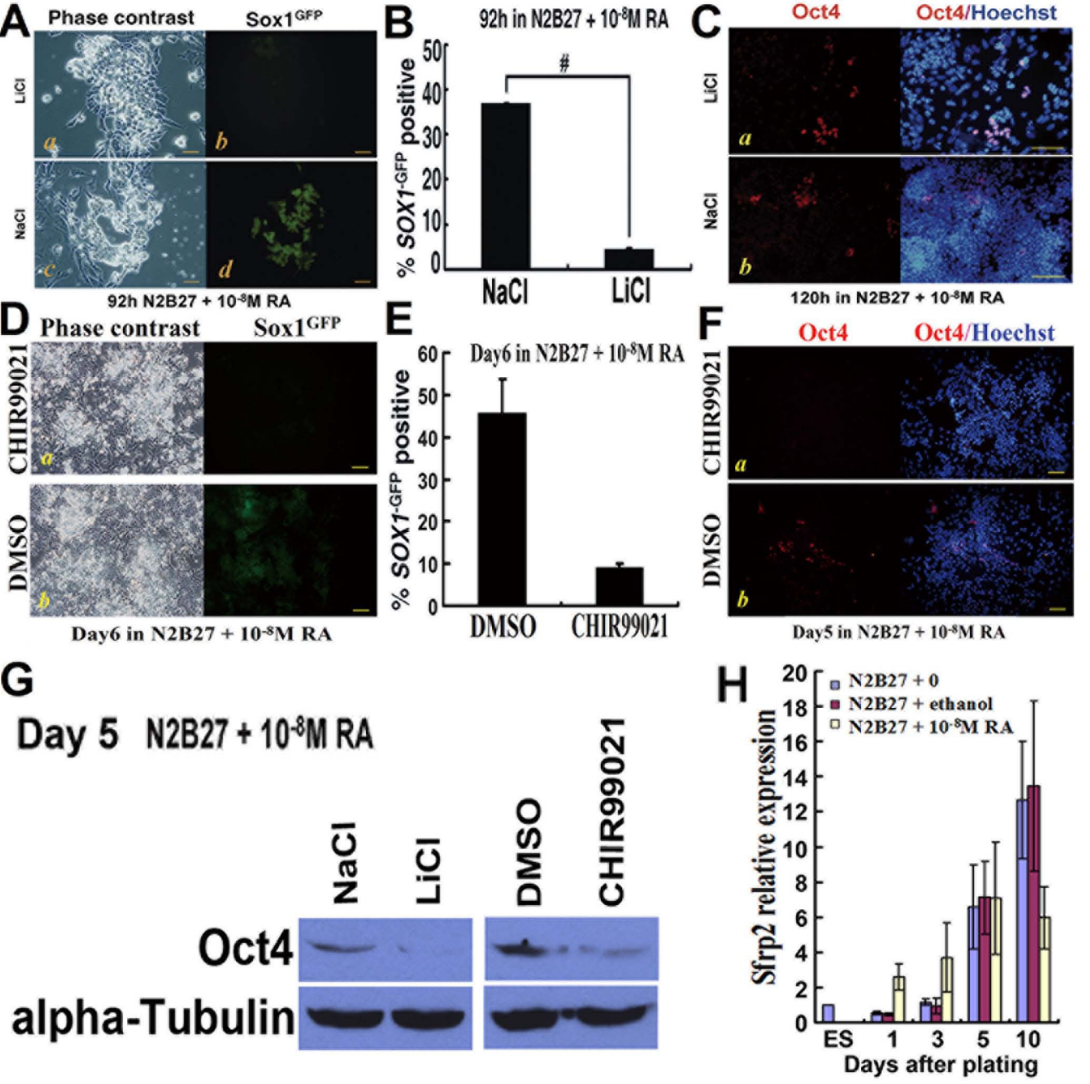
**Wnt signaling is involved in RA-promoted neural specification**. **A-C**, Monolayer differentiation of 46C ESCs in RA-treated cultures (N2B27 + 10^-8 ^M RA) exposed to the GSK-3 inhibitor LiCl (10 mM) or to NaCl (10 mM); **A**, Cultures of monolayer differentiation, shown in phase contrast or stained for *Sox1*^GFP^; **B**, Percentage of *Sox1*^GFP^+ cells scored by FACS. #, significant difference between inhibitor-treated cultures and NaCl-treated cultures (p < 0.001); **C**, Cultures of monolayer differentiation, shown in stained for Oct4 or combination of Oct4 and Hoechst; **D-F**, Monolayer differentiation of 46C ESCs in RA-treated cultures (N2B27 + 10^-8 ^M RA) exposed to the GSK-3 inhibitor CHIR99021 (3 μM) or to equivalent amounts of DMSO diluents; **D**, Cultures of monolayer differentiation, shown in phase contrast or stained for *Sox1*^GFP^; **E**, Percentage of *Sox1*^GFP^+ cells scored by FACS; **F**, Cultures of monolayer differentiation, shown in stained for Oct4 or combination of Oct4 and Hoechst; **G**, Western blot analysis of Oct4 at day 5 under monolayer cultures; **H**, Q-PCR for *Sfrp2 *during monolayer differentiation. Scale bars: A, 40 μm; C, D, F, 80 μm.

Wnt signaling has been implicated in regulating neural differentiation of ESCs. The secreted Wnt antagonist secreted frizzled-related protein 2 (Sfrp2) is up-regulated during neural differentiation of ESCs, and over-expression of Sfrp2 enhances neural differentiation. Activation of the Wnt pathway by treatment with lithium chloride (LiCl) inhibits neural differentiation of ESCs [25]. To investigate the effect of Wnt signaling, we added 10 mM LiCl to RA-treated cultures. Almost no GFP+ cells were found in LiCl and RA co-treated cultures, and most of the cells lost the Oct4 marker of ESCs (Figure 5A-C, G). CHIR99021, a more selective inhibitor of glycogen synthase kinase 3 (GSK3) [20,26], was also used. Similar results were gained (Figure 5D-G). Q-PCR for the expression of *Sfrp2 *showed that it was up-regulated at the early stage of differentiation (Figure 5H). The two GSK3 inhibitors also inhibited neural differentiation in the culture without RA (see Additional file 5 - Figure S5).

These findings showed that activation of Wnt signaling blocked the effect of RA. However, the roles of RA and signaling molecules involved in Wnt signaling still remain to be elucidated (see discussion).

## Discussion

The pluripotent ESC is a valuable *in vitro *model for studying the effects of various factors on cell lineage decisions in very early embryonic stages of mammalian development. In particular, the effects of RA signaling on the differentiation of ESCs and neural induction have been studied extensively. In addition to previous studies that have shown that RA promotes neural differentiation of ESCs [3,4,27], the present study demonstrated that RA facilitated the emergence of neural progenitors, neural stem cells and neurons in a monolayer culture. This is a simple system that allows direct observation, analysis and manipulation of the process of neural specification without the confounding influence of cell aggregation, co-culture, uncharacterized media constituents, or cell selection [9]. Furthermore, these results also demonstrated the novel and more precise molecular mechanisms of action of RA on neural differentiation.

### 1. Effects of RA on ESCs differentiation

RA and other retinoids are potent regulators of morphogenesis, growth and cell differentiation. RA, which is abundant in mammalian nervous systems [28], induces neurite outgrowth and neuronal differentiation from various sources, including ESCs [3,29], dorsal root ganglia [30], or neuroblastoma cells [31]. RA-induced neuronal differentiation is characterized by the expression of tissue-specific genes, proteins, ion channels, and receptors in a developmentally controlled manner [5].

Addition of RA promoted faster differentiation of ESCs, as indicated by the pattern of Oct4 expression, which was down-regulated more rapidly in RA-treated cultures, and could not be detected after day3. This is coincident with previous studies [32]. The expression levels of markers for undifferentiated neural cells, i.e., Sox1, *Pax6*, *Nestin*, *BLBP*, *Prominin*, *Olig2 *and *Musashi*, were higher in RA-treated cultures. As a consequence, the expression levels of markers for neurons, i.e., *Tuj1*, *MAP2*, and *NCAM*, and glial cells, i.e., *GFAP *were also elevated in RA-treated cultures. These findings are consistent with RA-induced differentiation of neural progenitor cells in the cell aggregation system, as described previously [3,27,33].

The initial higher frequency of *Sox1*^GFP^+ cells in RA-treated cultures is not caused mainly by the enhanced viability or proliferation of neural precursor cells. After treatment with RA, the number of purified *Sox1*^GFP^+ neural precursor cells decreased dramatically, which indicated that RA could not maintain the *Sox1*^GFP^+ state. This observation is coincident with the previous studies [34]. Treatment of adherent cultures with RA after the appearance of the first *Sox1*^GFP^+ cells increased the number of *Sox1*^GFP^+ cells. One explanation may be that, in monolayer culture, the speed at which RA induced ESCs into *Sox1*^GFP^+ neural precursor cells was faster than the speed of the loss of *Sox1*^GFP^+ cells.

### 2. Molecular mechanisms of action of RA on neural differentiation

#### 2.1 RA signaling is involved in efficient neural specification of ESCs

It is proposed that the molecular mechanisms of action of RA during embryogenesis *in vivo *involve a complex signaling pathway. RA exerts its function by binding to cellular RA-binding proteins that interact with the nuclear RA receptors. RA binds to RARs, which normally act as ligand-inducible transcription factors by binding as heterodimers with RXRs to RA response elements located in regulatory regions of target genes [35]. During *in vitro *differentiation of ESCs into the neuronal lineage, RA might act in the same way as it does *in vivo*. The hypothesis that *in vitro *and *in vivo *pathways are comparable is supported by significant up-regulation of *RARα *and *RARβ *mRNA, but rapid down-regulation of *RARγ *and *RXRγ *mRNA during RA-induced neuronal differentiation of mouse embryonal carcinoma cells [36]. This suggests a role for *RARα*, -*β*, -*γ *and *RXRγ *during neuroectodermal differentiation [37,38]. However, the mechanisms whereby RA induces neural differentiation of ESCs are not fully understood.

To demonstrate whether RA exerts its effects through RA-related nuclear receptors and to determine which receptors play more important roles in this efficient neural specification, we detected all six isoforms of RARs and RXRs by RT-PCR or Q-PCR. *RARα, RARγ *and *RXRβ *were detected in ESCs. During differentiation, few differences in the expression of *RARγ *and *RXRβ *were observed between the RA-treated and the two control groups, while obviously different expression of *RARα *was observed. In addition, the expression of *RARα *was up-regulated more at day 5 than at day 3. These observations hint that RARα may play an important role in RA-treated efficient neural specification. Adding the RARα-selective antagonist Ro 41-5253 effectively blocked the neural fate of ESCs, and many RA and Ro 41-5253 co-treated cells remained in an Oct4+ state. These findings suggest that RA signaling is indeed involved in promotion of this neural fate, and the nuclear receptor RARα plays a key role in this effect.

#### 2.2 RA activates the ERK pathway during promoted neural specification

Studies in planaria, frog embryos and avian embryos [17,18,39,40] have suggested a primary requirement for FGF signaling in neural specification. FGF4 is produced in an autocrine fashion by undifferentiated ESCs [41,42] and activates the Ras-MEK-ERK intracellular signaling cascades [22]. In our study, FGF receptor tyrosine kinase inhibitor SU5402 was used to try to block the neural specification effect of RA. This inhibitor could not significantly block the emergence of *Sox1*^GFP^+ neural progenitor cells in the RA-treated monolayer cultures, although it had the ability to inhibit the neural specification of ESCs when RA was absent from the culture ([7], see Additional file 6 - Figure S6). On the other hand, the two inhibitors PD184352 and PD0325901, both of which can block MEK1/2, upstream of ERK1/2, were able to block neural specification of ESCs even in the RA-treated cultures, and some cells remained as Oct4+ cells. All of these indicate that RA exerts its effects not through FGF signaling but requiring the activation of MEK-ERK signaling cascades. Previous studies have reported that RA can activate the ERK pathway in an unknown manner [43-46]. In our study, we also observed that phosphorylation of ERK was up-regulated at the early stage during differentiation of ESCs in RA-treated cultures, and the transcription factor *Elk-1*, one of the downstream targets of ERK [23,24], was also more up-regulated in the RA-treated cultures than in the two control cultures before day3. Activated ERK1/2 also mediates phosphorylation of the MAPK sites of Smad1 (pSmad1^MAPK^), which inhibits the inhibitory function of the bone morphogenetic proteins (BMP)-Smad1 pathway on neural differentiation [47].

**Figure 6:**
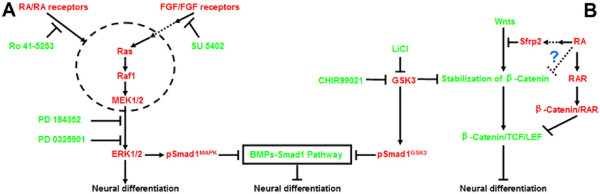
**A. Interaction between RA and the ERK pathway during promoted neural specification**. FGF receptor tyrosine kinase inhibitor SU5402 and MEK1/2 inhibitors PD184352 and PD0325901, respectively suppresses neural differentiation of ESCs. In RA-treated cultures, PD184352, PD0325901 and RARα-selective antagonist Ro 41-5253 could inhibit the neural specification of ESCs, while SU5402 shows weak neural inhibition effect. RA probably enhances ERK phosphorylation via MEK1/2 or its upstream but not in a manner that is dependent on FGF signaling, and activated ERK1/2 mediates phosphorylation of the MAPK sites of Smad1 (pSmad1^MAPK^), which inhibits the inhibitory function of the bone morphogenetic proteins (BMPs)-Smad1 pathway on neural differentiation. Blockade of RA signaling might affect the activation of the upstream of ERK1/2, which leads to failure of neural specification of ESCs even with the stimulation of FGF signaling. **B. Possible roles of Wnt signaling in RA-promoted neural differentiation**. RA promotes neural specification probably by up-regulating Sfrp2, which inhibits the Wnt-β-catenin anti-neural pathway. Upon adding LiCl or CHIR99021, which stimulates the β-catenin signaling and/or BMP signaling through inhibition of GSK3, the suppression effects on neural specification of β-catenin pathway can be passed down even with the stimuli of RA. RA also may promote neural differentiation by up-regulating RAR, which may decrease β-catenin/LEF/TCF mediated transactivation. There exists a possibility that RA may suppress the inhibition effects on neural differentiation of Wnt-β-catenin pathway by sequestration of β-catenin. pSmad1^GSK3 ^indicates phosphorylation of the GSK3 sites of Smad1.

Based on the above, we propose that one possible mechanism of how RA promotes neural lineage entry by ESCs is that it enhances ERK phosphorylation via MEK1/2 or its upstream but not via a mechanism that is dependent on FGF signaling, and blockade of RA signaling may affect the activation of the upstream of ERK1/2, which leads to failure of neural specification of ESCs, even with the stimulation of FGF signaling (Figure 6A).

#### 2.3 Wnt signaling also plays a role in RA-promoted neural differentiation

Down-regulation of Wnt signaling has been shown to be one of the mechanisms involved in RA-induced neural differentiation of mouse ESCs. Up-regulation of Sfrp2 can inhibit the Wnt-β-catenin anti-neural pathway. Conversely, adding LiCl, which inhibits GSK3 and partially mimics Wnt signaling, can enhance the anti-neural pathway [25]. On the other hand, GSK3 mediated-Smad1 phosphorylation can block the inhibitory effect of BMPs on neural differentiation [47]. Therefore, inhibition of GSK3 may also make the BMP-Smad1 pathway become valid for inhibiting neural differentiation. Interestingly, β-catenin, which is a key molecule in Wnt signaling, has been shown to interact directly with RAR, and as a result retinoids decrease activation of T-cell transcription factor (TCF) in cultured cells in a dose-dependent manner [48].

In the present study, we found that RA up-regulated the expression of RARα and RARβ at the early stage of differentiation. We also investigated the effect of LiCl and another GSK3 inhibitor CHIR99021. Negative effects of Wnt pathways on neural differentiation were observed by adding the two inhibitors to RA-treated cultures, and most cells lost the marker of ESCs. RA also showed its effect on up-regulating the expression of Sfrp2.

Based on the present and previous studies, RA may exert its effects through the following mechanisms (Fig. 6B). (1) RA promotes neural specification by up-regulating Sfrp2. The expression of Sfrp2 is up-regulated by stimulation with RA, which blocks the message passing downstream from Wnts [25]. Upon adding LiCl or CHIR99021, which stimulates β-catenin and/or BMP signaling through inhibition of GSK3, but not through Wnts, the β-catenin pathway and/or BMP signaling can still play a part in suppressing neural specification, even with the stimulus of RA-up-regulated Sfrp2. (2) RA may promote neural differentiation by up-regulating RAR, which may decrease β-catenin-lymphoid enhancing factor (LEF)/TCF mediated transactivation. (3) RA also may enhance the sequestration of β-catenin to suppress the anti-neural effects of the Wnt-β-catenin pathway. However, this hypothesis needs further evidence.

## Conclusion

Our study showed that RA can promote neural lineage entry by ESCs in adherent monolayer culture systems. We also found that RA stimulation led to dramatic changes in RA nuclear receptor and RA-metabolizing enzyme expression. Further study showed that these effects of RA depended on RA signaling and its crosstalk with the ERK and Wnt pathways. Our study showed a more limpid circumscription of the effects of RA and its pathway on neural lineage choice of mouse ESCs.

## Methods

### ESC culture

Mouse ESC line 46C (passage 25-35), which has one allele of *Sox1 *inactivated by targeted integration of a GFPirespac cassette, was cultivated as previously described [49]. Briefly, ESCs were maintained on gelatin-coated dishes in the absence of feeder cells in GMEM (Sigma, St. Louis, MO) supplemented with 2 mM glutamine (Gibco, Grand Island, NY), 0.001% β-mercaptoethanol (Gibco), 1× nonessential amino acids (Gibco), 10% fetal bovine serum (FBS) (Gibco), and 2,000 units/ml human recombinant LIF (R & D Systems, Minneapolis, MN).

### ESC adherent monoculture

Procedure was described in detail in [7]. Briefly, ESCs were washed with N2B27 serum-free medium to remove serum and then plated onto 0.1% gelatin-coated tissue culture plastic at a density of 1 × 10^4 ^cells/cm^2 ^in N2B27 with or without RA (Sigma). N2B27 consists of a 1:1 ratio of DMEM/F12 and Neurobasal media supplemented with 0.5% modified N2 (Gibco), 0.5% B27 (Gibco), and 2-mercaptoethanol (Gibco). Medium was changed every other day. For purifying *Sox1*^GFP^+ cells, 0.5 μg/ml puromycin (Sigma) was used.

For clonal cultures assay, dissociated ESCs were plated onto 0.1% gelatin-coated 6-well plates (Corning Incorporated, Corning, NY) at the density of 1.5 × 10^3 ^cells/well and cultured in serum-containing ESC medium (3 ml/well) plus LIF. At this density, more than 90% of colonies derived from single cells. After 2 days, medium was replaced with serum-free neural differentiation medium N2B27. Medium was renewed with fresh N2B27 every other day. After 2 days culture in N2B27 medium, total colonies and colonies containing *Sox1*^GFP^+ cells were counted.

Cells were counted by using a hemocytometer.

### Immunofluorescence and FACS

Cells were fixed in 4% paraformaldehyde and incubated for 1 h in blocking buffer (PBS, 10% FBS, and 0.1% Triton X-100). Primary antibodies were diluted in blocking buffer and applied overnight at 4°C. After three washes in PBS, secondary antibodies conjugated to TRITC (tetramethylrhodamine isothiocyanate) (Vector, Burlingame, CA) were diluted at 1: 200 in blocking buffer and applied for 1 h at room temperature. The cells were washed at least three times in PBS and visualized on an Olympus inverted fluorescence microscope. For nuclear counter staining, nuclei were stained with bisbenzimide (Hoechst 33342, 10 μM, Sigma).

Primary antibodies were obtained from the following sources: Oct4 (1: 500, Santa Cruz Biotechnology, Santa Cruz, CA), nestin (1:200, Chemicon, Temecula, CA), Tuj1 (1:200, R & D Systems), MAP2 (1: 200, Sigma), GFAP (1:400, Chemicon).

FACS analysis was performed using a Becton-Dickinson (Palo Alto, CA) FACS Calibur flow cytometer.

### RT-PCR and Q-PCR analysis

RT-PCR analysis of at least three independent cultures was performed in most of the experiments, and similar results were obtained.

RNA was isolated using TRIzol (Invitrogen, Carlsbad, CA), DNase I-digested (Invitrogen), and column-purified (RNeasy MinElute Cleanup; Qiagen, Valencia, CA). First-strand cDNA was synthesized using M-MLV Reverse Transcriptase (Invitrogen). To analyze relative expression of different mRNAs, the amount of cDNA was normalized based on the signals from ubiquitously expressed *GAPDH *mRNA. To provide negative controls and exclude contamination by genomic DNA, the reverse transcriptase was omitted in the cDNA synthesis step, and the samples were subjected to the PCR reaction in the same manner with primer sets for *GAPDH*, and were indicated at the bottom of each figure as RT (-). Primer sequences and PCR cycling conditions could be found in the Additional file 7 - Table S1.

Q-PCR was performed with Power SYBR Green PCR Master Mix (Applied Biosystems, Foster City, CA) according to manufacturer's instructions. Signals were detected with an ABI7300 Real-Time PCR System (Applied Biosystems). The relative expression level was determine by the 2^-delta Ct ^method and normalized against ribosomal protein L19 (RPL19). Primer sequences could be found in the Additional file 8 - Table S2.

### Western blot analysis

Cells and tissues were lysed in a lysis buffer (20 mM Tris-HCl, pH 7.4, 2% Triton X-100, 10 mM EDTA, 5 mM NaF and 1 mM sodium orthovanadate). Protein concentration was determined by BCA assay (Pierce, Rockford, IL). Then proteins were resolved in 12% SDS polyacrylamide gels and transferred onto nitrocellulose membranes. The membranes were then incubated overnight at 4°C in blocking solution containing 5% nonfat dry milk in PBS with 0.1% Tween-20. Subsequently the membranes were incubated with primary antibody against Oct-4 (1:1000, Santa Cruz), ERK1/2, phosphor-ERK1/2 (both 1:500, Cell Signaling Technology, MA), Neuronal Nuclei (NeuN, 1:200, Chemicon), β-actin (1:2000, Biovision, Mountain View, CA) or alpha Tubulin (1:2000, Sigma) followed by incubation with HRP (horseradish peroxidase)-conjugated second antibodies. Detection of HRP was performed by SuperSignal West Pico Chemiluminescent Substrate (Pierce).

### Pharmacological reagents

Ro 41-5253 (Biomol, Plymouth Meeting, PA) was used at 1 μM. PD184352, PD0325901 and CHIR99021 (kind gifts of Professor Qilong Ying, University of Southern California, USA) was used at a concentration of 4 μM, 4 μM and 3 μM respectively. SU5402 (Calbiochem, San Diego, CA) was used at 5 μM. LiCl (Sigma) was used at 10 mM.

### Data analysis

The statistics were analyzed by STAT 8.0 software. For comparisons of two conditions, the Student's *t *test was used. All data were expressed as mean ± standard error of mean (S.E.M.).

## Abbreviations

BLBP: (brain lipid binding protein); ERK: extracellular signal-regulated kinase; ESCs: embryonic stem cells; FGF: fibroblast growth factor; FBS: fetal bovine serum; GAPDH: glyceraldehyde-3-phosphate dehydrogenase; GFAP: glial fibrillary acidic protein; GFP: green fluorescent protein; GSK3: glycogen synthase kinase; HRP: horseradish peroxidase; LEF: lymphoid enhancing factor; LiCl: lithium chloride; LIF: leukemia inhibitory factor; MAP2: microtubule associated protein 2; MEK: MAP kinase/ERK kinase; NCAM: neural cell adhesion molecule; NeuN: Neuronal Nuclei; RA: alltrans retinoic acid; RAR: retinoic acid receptor; RXR: retinoid × receptor; Sfrp2: secreted frizzled-related protein 2; TCF: T-cell transcription factor; TRITC: tetramethylrhodamine isothiocyanate.

## Authors' contributions

JFL Designed the study, performed all experiments and drafted the manuscript; LT Performed cell culture and immunofluorescence analysis; PL Performed cell culture; HG Performed FACS analysis; BF Performed FACS analysis; SDY Performed RT-PCR; ZG Performed western blot; PZ Contributed to manuscript draft; HYS Designed the study and drafted the manuscript; All authors read and approved the final manuscript.

## Supplementary Material

Additional file 1 **High does of RA (10^-6 ^M) shows obvious noxious effect on cell survival**. ESCs were cultured under monolayer differentiation conditions with low does of RA (N2B27 + 10^-8 ^M RA), high does of RA (10^-6 ^M) or without RA (N2B27 + 0 or N2B27 + ethanol). **A**, Intact cultures at different time points of monolayer differentiation, shown in phase contrast; **B**, Ratios of harvested cell number to plated cell number at day 10 (average of triplicates). Click here for file

Additional file 2 **RA increases the cell number of the monolayer culture at the early stage of neural induction**. Ratios of harvested cell number to plated cell number at various time points were given (average of triplicates). Click here for file

Additional file 3 **RA shows less effect on neural differentiation when ESCs were plated at a high density (3 × 10^4 ^cells/cm^2^)**. Proportion of *Sox1*^GFP^+ cells at various time points was given (average of triplicates). Click here for file

Additional file 4 **Effects of inhibitors on cell viability or proliferation in RA-treated monolayer cultures**. Monolayer differentiation of ESCs in RA-treated cultures (N2B27 + 10^-8 ^M RA) exposed to FGF receptor tyrosine kinase inhibitor SU5402 (5 μM), MEK1/2 inhibitors PD184352 (4 μM) or PD0325901 (4 μM), or to equivalent amounts of DMSO diluents. Ratios of harvested cell number to plated cell number at 92 h were given (average of triplicates). Click here for file

Additional file 5 **GSK3 inhibitors block the neural specification of ESCs when RA is absent in the culture**. A, B, LiCl inhibits the neural specification of ESCs when RA is absent in the culture. Monolayer differentiation of 46C ESCs in cultures without RA (N2B27) exposed to LiCl (10 mM) or to NaCl (10 mM) at day 4. A, Cultures of monolayer differentiation, shown in phase contrast or stained for Sox1GFP. B, Percentage of Sox1GFP+ cells scored by FACS. C, D, CHIR99021 inhibits the neural specification of ESCs when RA is absent in the culture. Monolayer differentiation of 46C ESCs in cultures without RA (N2B27) exposed to the GSK3 inhibitor CHIR99021 (3 μM) or to equivalent amounts of DMSO diluents. C, Cultures of monolayer differentiation, shown in phase contrast or stained for Sox1GFP. D, Percentage of Sox1GFP+ cells scored by FACS. Scale bars: A, C, 80 μm. Click here for file

Additional file 6 **SU 5402 inhibits the neural specification of ESCs when RA is absent in the culture**. Monolayer differentiation of 46C ESCs in cultures without RA (N2B27 + 0) exposed to FGF receptor tyrosine kinase inhibitor SU5402 (5 μM) or to equivalent amounts of DMSO diluents at day 6. **A**, Cultures of monolayer differentiation, shown in phase contrast or stained for *Sox1*^GFP^. **B**, Typical FACS profile of *Sox1*^GFP ^expression. **C**, Percentage of *Sox1*^GFP^+ cells scored by FACS. Scale bars: A, 80 μm. Click here for file

Additional file 7 RT-PCR primer specifications. Click here for file

Additional file 8 Q-PCR primer specifications. Click here for file

## References

[B1] Dani C, Smith AG, Dessolin S, Leroy P, Staccini L, Villageois P, Darimont C, Ailhaud G (1997). Differentiation of embryonic stem cells into adipocytes *in vitro*. J Cell Sci.

[B2] Maden M (2007). Retinoic acid in the development, regeneration and maintenance of the nervous system. Nat Rev Neurosci.

[B3] Bain G, Kitchens D, Yao M, Huettner JE, Gottlieb DI (1995). Embryonic stem cells express neuronal properties *in vitro*. Dev Biol.

[B4] Fraichard A, Chassande O, Bilbaut G, Dehay C, Savatier P, Samarut J (1995). *In vitro *differentiation of embryonic stem cells into glial cells and functional neurons. J Cell Sci.

[B5] Guan K, Chang H, Rolletschek A, Wobus AM (2001). Embryonic stem cell-derived neurogenesis: Retinoic acid induction and lineage selection of neuronal cells. Cell Tissue Res.

[B6] Gordon MK (1995). *In vitro *differentiation of embryonic stem cells. Curr Opi Cell Biol.

[B7] Ying QL, Stavridis M, Griffiths D, Li M, Smith AG (2003). Conversion of embryonic stem cells into neuroectodermal precursors in adherent monoculture. Nat Biotechnol.

[B8] Marill J, Idres N, Capron CC, Nguyen E, Chabot GG (2003). Retinoic acid metabolism and mechanism of action: a review. Curr Drug Metab.

[B9] Ying QL, Smith AG (2003). Defined conditions for neural commitment and differentiation. Methods Enzymol.

[B10] Mountford P, Zevnik B, Düwel A, Nichols J, Li M, Dani C, Robertson M, Chambers I, Smith A (1994). Dicistronic targeting constructs: Reporters and modifiers of mammalian gene expression. Proc Natl Acad Sci USA.

[B11] Wood HB, Episkopou V (1999). Comparative expression of the mouse Sox1, Sox2 and Sox3 genes from pre-gastrulation to early somite stages. Mech Dev.

[B12] Niwa H, Miyazaki J, Smith AG (2000). Quantitative expression of Oct-3/4 defines differentiation, dedifferentiation or self-renewal of ESCs. Nat Genet.

[B13] Takebayashi H, Yoshida S, Sugimori M, Kosako H, Kominami R, Nakafuku M, Nabeshima Y (2000). Dynamic expression of basic helix-loop-helix Olig family members: implication of Olig2 in neuron and oligodendrocyte differentiation and identification of a new member, Olig3. Mech Dev.

[B14] Apfel C, Bauer F, Crettaz M, Forni L, Kamber M, Kaufmann F, LeMotte P, Pirson W, Klaus M (1992). A retinoic acid receptor alpha antagonist selectively counteracts retinoic acid effects. Proc Natl Acad Sci USA.

[B15] Streit A, Berliner AJ, Papanayotou C, Sirulnik A, Stern CD (2000). Initiation of neural induction by FGF signaling before gastrulation. Nature.

[B16] Wilson SI, Graziano E, Harland R, Jessell TM, Edlund T (2000). An early requirement for FGF signaling in the acquisition of neural cell fate in the chick embryo. Curr Biol.

[B17] Pera EM, Ikeda A, Eivers E, De Robertis EM (2003). Integration of IGF, FGF, and anti-BMP signals via Smad1 phosphorylation in neural induction. Genes Dev.

[B18] Mohammadi M, McMahon G, Sun L, Tang C, Hirth P, Yeh BK, Hubbard SR, Schlessinger J (1997). Structures of the tyrosine kinase domain of fibroblast growth factor receptor in complex with inhibitors. Science.

[B19] Dai Y, Yu C, Singh V, Tang L, Wang Z, McInistry R, Dent P, Grant S (2001). Pharmacological inhibitors of the mitogen-activated protein kinase (MAPK) kinase/MAPK cascade interact synergistically with UCN-01 to induce mitochondrial dysfunction and apoptosis in human leukemia cells. Cancer Res.

[B20] Bain J, Plater L, Elliott M, Shpiro N, Hastie CJ, Mclauchlan H, Klevernic I, Arthur JS, Alessi DR, Cohen P (2007). The selectivity of protein kinase inhibitors: a further update. Biochem J.

[B21] Stavridis MP, Smith AG (2003). Neural differentiation of mouse embryonic stem cells. Biochem Soc Trans.

[B22] Ying QL, Wray J, Nichols J, Batlle-Morera L, Doble B, Woodgett J, Cohen P, Smith AG (2008). The ground state of embryonic stem cell self-renewal. Nature.

[B23] Marais R, Wynne J, Treisman R (1993). The SRF accessory protein Elk-1 contains a growth factor-regulated transcriptional activation domain. Cell.

[B24] Kortenjann M, Thomae O, Shaw PE (1994). Inhibition of v-raf-dependent c-fos expression and transformation by a kinase-defective mutant of the mitogen-activated protein kinase Erk2. Mol Cell Biol.

[B25] Aubert J, Dunstan H, Chambers I, Smith A (2002). Functional gene screening in embryonic stem cells implicates Wnt antagonism in neural differentiation. Nat Biotechnol.

[B26] Murray JT, Campbell DG, Morrice N, Auld GC, Shpiro N, Marquez R, Peggie M, Bain J, Bloomberg GB, Grahammer F, Lang F, Wulff P, Kuhl D, Cohen P (2004). Exploitation of KESTREL to identify NDRG family members as physiological substrates for SGK1 and GSK3. Biochem J.

[B27] Bain G, Ray WJ, Yao M, Gottlieb DI (1996). Retinoic acid promotes neural and represses mesodermal gene expression in mouse embryonic stem cells in culture. Biochem Biophys Res Commun.

[B28] Maden M (2001). Role and distribution of retinoic acid during CNS development. Int Rev Cytol.

[B29] Li M, Pevny L, Lovell-Badge R, Smith A (1998). Generation of purified neural precursors from embryonic stem cells by lineage selection. Curr Biol.

[B30] Corcoran J, Maden M (1999). Nerve growth factor acts via retinoic acid synthesis to stimulate neurite outgrowth. Nat Neurosci.

[B31] Haussler M, Sidell N, Kelly M, Donaldson C, Altman A, Mangelsdorf D (1983). Specific high affinity binding and biological action of retinoic acid in human neuroblastoma cells. Proc Natl Acad Sci USA.

[B32] Schoorlemmer J, Jonk L, Sanbing S, van Puijenbroek A, Feijen A, Kruijer W (1995). Regulation of Oct-4 gene expression during differentiation of EC cells. Mol Biol Rep.

[B33] Martín-Ibáñez R, Urbán N, Sergent-Tanguy S, Pineda JR, Garrido-Clua N, Alberch J, Canals JM (2007). Interplay of leukemia inhibitory factor and retinoic acid on neural differentiation of mouse embryonic stem cells. J Neurosci Res.

[B34] Takahashi J, Palmer TD, Gage FH (1999). Retinoic Acid and Neurotrophins Collaborate to Regulate Neurogenesis in Adult-Derived Neural Stem Cell Cultures. J Neurobiol.

[B35] Chambon P (1996). A decade of molecular biology of retinoic acid receptors. FASEB J.

[B36] Gottlieb DI, Huettner JE (1999). An *in vitro *pathway from embryonic stem cells to neurons and glia. Cells Tissues Organs.

[B37] Jonk LJ, Jonge ME de, Kruyt FA, Mummery CL, Saag PT, Kruijer W van der (1992). Aggregation and cell cycle dependent retinoic acid receptor mRNA expression in P19 embryonal carcinoma cells. Mech Dev.

[B38] Yokota Y, Ohkubo H (1996). 9-*cis*-Retinoic acid induces neuronal differentiation of retinoic acid-nonresponsive embryonal carcinoma cells. Exp Cell Res.

[B39] Cebrià F, Kobayashi C, Umesono Y, Nakazawa M, Mineta K, Ikeo K, Gojobori T, Itoh M, Taira M, Sánchez Alvarado A, Agata K (2002). FGFR-related gene *nou-darake *restricts brain tissues to the head region of planarians. Nature.

[B40] Launay C, Fromentoux V, Shi DL, Boucaut JC (1996). A truncated FGF receptor blocks neural induction by endogenous *Xenopus *inducers. Development.

[B41] Ma YG, Rosfjord E, Huebert C, Wilder P, Tiesman J, Kelly D, Rizzino A (1992). Transcriptional regulation of the murine k-FGF gene in embryonic cell lines. Dev Biol.

[B42] Rathjen J, Lake JA, Bettess MD, Washington JM, Chapman G, Rathjen PD (1999). Formation of a primitive ectoderm like cell population, EPL cells, from ESCs in response to biologically derived factors. J Cell Sci.

[B43] Kampmann E, Mey J (2007). Retinoic acid enhances Erk phosphorylation in the chick retina. Neurosci Lett.

[B44] Cañón E, Cosgaya JM, Scsucova S, Aranda A (2004). Rapid effects of retinoic acid on CREB and ERK phosphorylation in neuronal cells. Mol Biol Cell.

[B45] Bost F, Caron L, Marchetti I, Dani C, Le Marchand-Brustel Y, Binétruy B (2002). Retinoic acid activation of the ERK pathway is required for embryonic stem cell commitment into the adipocyte lineage. Biochem J.

[B46] Li Z, Theus MH, Wei L (2006). Role of ERK 1/2 signaling in neuronal differentiation of cultured embryonic stem cells. Develop Growth Differ.

[B47] Fuentealba LC, Eivers E, Ikeda A, Hurtado C, Kuroda H, Pera EM, De Robertis EM (2007). Integrating patterning signals: Wnt/GSK3 regulates the duration of the BMP/Smad1 signal. Cell.

[B48] Easwaran V, Pishvaian M, Salimuddin S, Byers S (1999). Crossregulation of [beta]-catenin-LEF/TCF and retinoid signaling pathways. Curr Biol.

[B49] Aubert J, Stavridis MP, Tweedie S, O'Reilly M, Vierlinger K, Li M, Ghazal P, Pratt T, Mason JO, Roy D, Smith A (2003). Screening for mammalian neural genes via fluorescence-activated cell sorter purification of neural precursors from Sox1-gfp knock-in mice. Proc Natl Acad Sci USA.

