# Supplementary figures and images for: All-trans retinoic acid promotes neural lineage entry by pluripotent embryonic stem cells via multiple pathways
Source: BMC Cell Biol. 2009 Jul 30;10:57. doi: 10.1186/1471-2121-10-57 (PMC2728515; doi:10.1186/1471-2121-10-57)

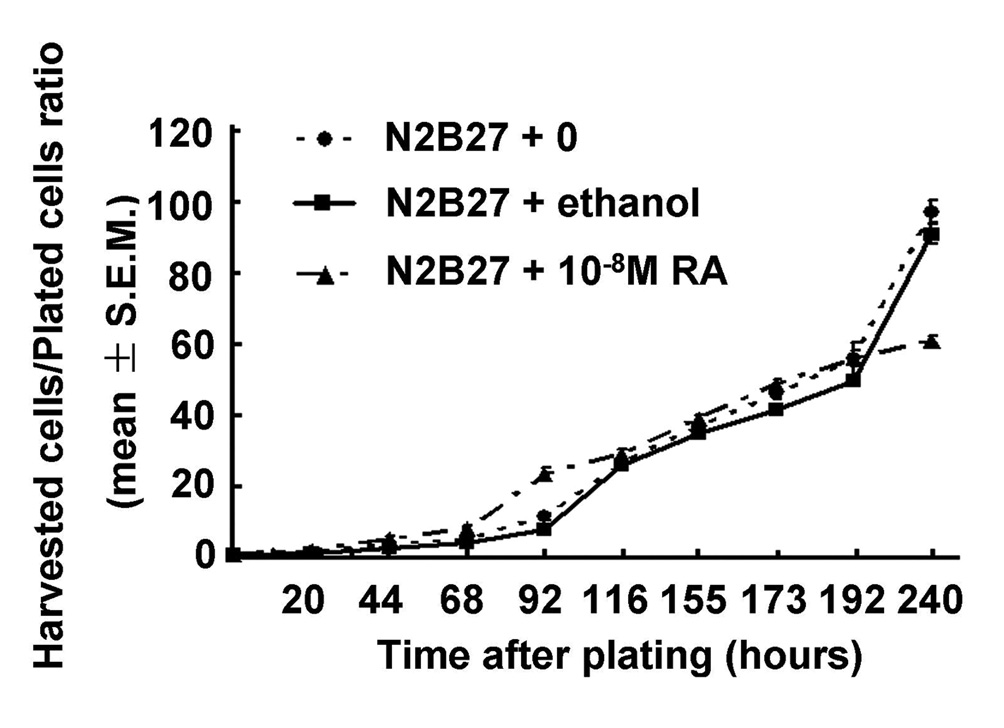

Supplement: Additional file 2 — RA increases the cell number of the monolayer culture at the early stage of neural induction. Ratios of harvested cell number to plated cell number at various time points were given (average of triplicates). [file 1471-2121-10-57-S2.tiff]

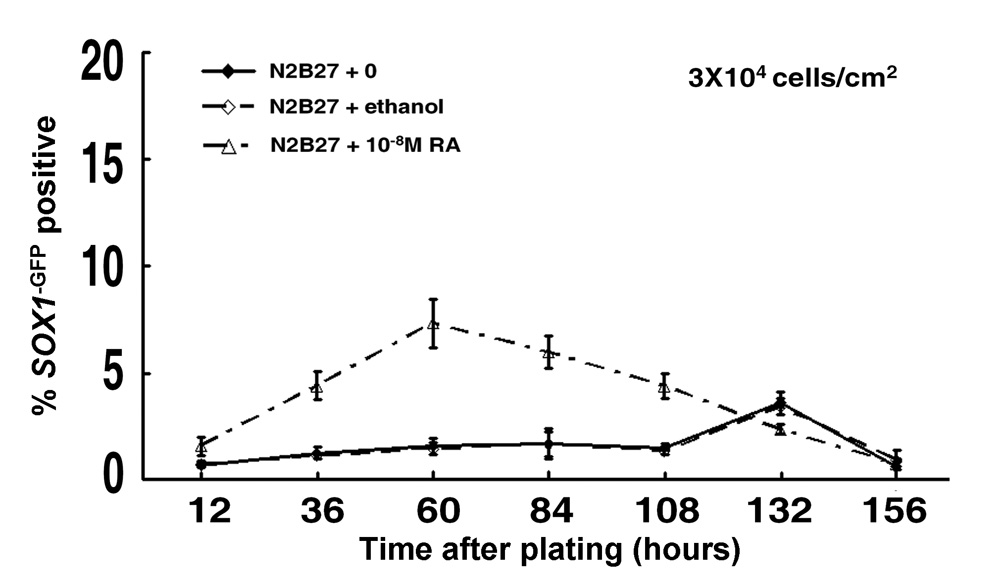

Supplement: Additional file 3 — RA shows less effect on neural differentiation when ESCs were plated at a high density (3 × 10^4^ cells/cm2). Proportion of Sox1GFP+ cells at various time points was given (average of triplicates). [file 1471-2121-10-57-S3.tiff]

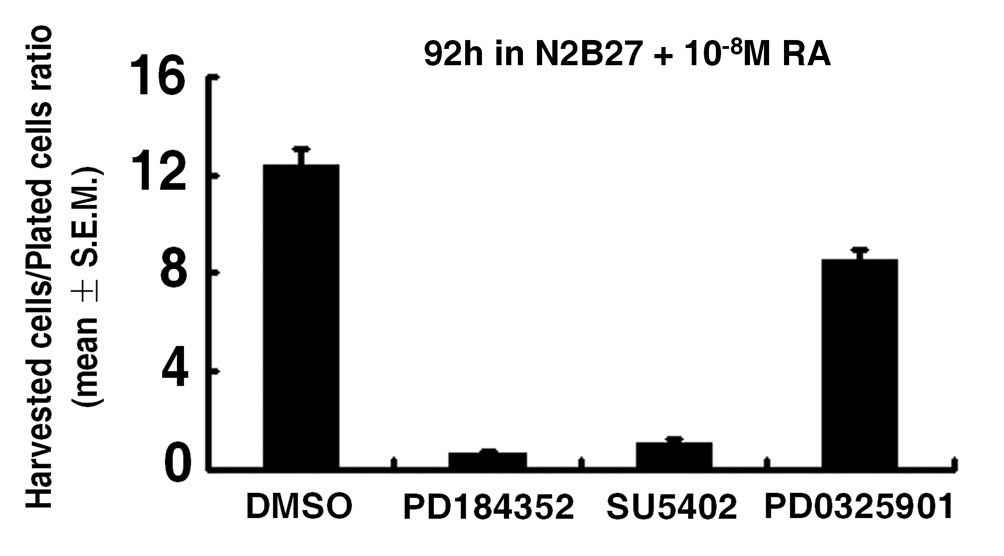

Supplement: Additional file 4 — Effects of inhibitors on cell viability or proliferation in RA-treated monolayer cultures. Monolayer differentiation of ESCs in RA-treated cultures (N2B27 + 10-8 M RA) exposed to FGF receptor tyrosine kinase inhibitor SU5402 (5 μM), MEK1/2 inhibitors PD184352 (4 μM) or PD0325901 (4 μM), or to equivalent amounts of DMSO diluents. Ratios of harvested cell number to plated cell number at 92 h were given (average of triplicates). [file 1471-2121-10-57-S4.tiff]
