# Supplementary material for: All-trans retinoic acid promotes neural lineage entry by pluripotent embryonic stem cells via multiple pathways
Source: BMC Cell Biol. 2009 Jul 30;10:57. doi: 10.1186/1471-2121-10-57 (PMC2728515; doi:10.1186/1471-2121-10-57)
Supplement: Additional file 7 — RT-PCR primer specifications. [file 1471-2121-10-57-S7.doc]

Table S1: PCT Primer Specification.

| **Gene** | **Accession no.** | **Forward primer** | **Reverse primer** | **Size(bp)** | **Cycles** | **Parameters** |  |
| --- | --- | --- | --- | --- | --- | --- | --- |
| Oct3/4 | [NM_013633](http://www.ncbi.nlm.nih.gov/entrez/viewer.fcgi?db=nuccore&val=125490391) | 5'GCTCAGCCTTAAGAACATGTGTAAGC3' | 5'GCCTCATACTCTTCTCGTTGGGA3' | 327 | 24 | 94℃-40 seconds; 60℃-30 seconds; 72℃-30 seconds |  |
| Pax6 | [NM_013627](http://www.ncbi.nlm.nih.gov/entrez/viewer.fcgi?db=nuccore&val=146134521) | 5'GAAATCCGAGACAGATTATTATCCGAG3' | 5'CCATTTGGCCCTTCGATTAGA3' | 495 | 25 | 94℃-40 seconds; 58℃-30 seconds; 72℃-40 seconds |  |
| Nestin | [NM_016701](http://www.ncbi.nlm.nih.gov/entrez/viewer.fcgi?db=nucleotide&val=50363231) | 5'AGTCAGAGCAAGTGAATGG3' | 5'AGAAACAAGATCTCAGCAGG3' | 600 | 30 | 94℃-40 seconds; 55℃-30 seconds; 72℃-40 seconds |  |
| BLBP | [S69799](http://www.ncbi.nlm.nih.gov/entrez/viewer.fcgi?db=nucleotide&val=546458) | 5'TGAGTACATGAAAGCTCTGGGCGT3' | 5'TGAGCTTGTCTCCATCCAACCGAA3' | 224 | 30 | 94℃-40 seconds; 58℃-30 seconds; 72℃-30 seconds |  |
| Prominin | [NM_008935](http://www.ncbi.nlm.nih.gov/entrez/viewer.fcgi?db=nucleotide&val=6679478) | 5'GAGTCCTTATCTGCGCCATC3' | 5'GTCCTGGTCTGCTGGTTAGC3' | 232 | 27 | 94℃-40 seconds; 55℃-30 seconds; 72℃-30 seconds |  |
| Olig2 | [NM_016967](http://www.ncbi.nlm.nih.gov/entrez/viewer.fcgi?db=nucleotide&val=59858556) | 5'GGCGGTGGCTTCAAGTCATC3' | 5'TAGTTTCGCGCCAGCAGCAG3' | 265 | 35 | 94℃-40 seconds; 58℃-30 seconds; 72℃-30 seconds |  |
| Musashi | [NM_008629](http://www.ncbi.nlm.nih.gov/entrez/viewer.fcgi?db=nuccore&val=6678939) | 5'CACGACCCCTGCAAGATGTTC3' | 5'CCATCTTAGGCTGTGCTCTTCGA3' | 260 | 35 | 94℃-40 seconds; 62℃-30 seconds; 72℃-30 seconds |  |
| Tuj1 | [NM_023279](http://www.ncbi.nlm.nih.gov/entrez/viewer.fcgi?db=nucleotide&val=31982671) | 5'ATCCACCTTCATTGGCAACAGCAC3' | 5'ACTCGGACACCAGGTCATTCATGT3' | 173 | 28 | 94℃-40 seconds; 58℃-30 seconds; 72℃-30 seconds |  |
| MAP2 | [M21041](http://www.ncbi.nlm.nih.gov/entrez/viewer.fcgi?db=nucleotide&val=199022) | 5'AGCCGCAACGCCAATGGATT3' | 5'TTTGTTCCGAGGCTGGCGAT3' | 313 | 25 | 94℃-40 seconds; 60℃-30 seconds; 72℃-30 seconds |  |
| NCAM | [NM_010875](http://www.ncbi.nlm.nih.gov/entrez/viewer.fcgi?db=nucleotide&val=124517682) | 5'TTCCTGTGTCAAGTGGCAGGAGAT3' | 5'AGATCTTCACGTTGACAGTGGCCT3' | 229 | 26 | 94℃-40 seconds; 60℃-30 seconds; 72℃-30 seconds |  |
| RARα | [NM_009024](http://www.ncbi.nlm.nih.gov/entrez/viewer.fcgi?db=nucleotide&val=116734872) | 5'CTTCTGACTGTGGCTGCTTG3' | 5'CTCTTCGGAACTGCTGCTCT3' | 232 | 35 | 94℃-40 seconds; 60℃-30 seconds; 72℃-30 seconds |  |
| RARβ | [NM_011243](http://www.ncbi.nlm.nih.gov/entrez/viewer.fcgi?db=nucleotide&val=45593123) | 5'GGACCTTGAGGAACCAACAA3' | 5'GAATGTCTGCAACAGCTGGA3' | 375 | 35 | 94℃-40 seconds; 55℃-30 seconds; 72℃-30 seconds |  |
| RARγ | [NM_001042727](http://www.ncbi.nlm.nih.gov/entrez/viewer.fcgi?db=nucleotide&val=112181195) | 5'AGGTCACCAGAAATCGATGC3' | 5'CTGGCAGAGTGAGGGAAAAG3' | 212 | 28 | 94℃-40 seconds; 55℃-30 seconds; 72℃-30 seconds |  |
| RXRα | [NM_011305](http://www.ncbi.nlm.nih.gov/entrez/viewer.fcgi?db=nucleotide&val=84579898) | 5'CTTTGACAGGGTGCTAACAGAGC3' | 5'ACGCTTCTAGTGACGCATAACACC3' | 173 | 35 | 94℃-40 seconds; 57℃-30 seconds; 72℃-30 seconds |  |
| RXRβ | [NM_011306](http://www.ncbi.nlm.nih.gov/entrez/viewer.fcgi?db=nucleotide&val=118130198) | 5'TCAACTCCACAGTGTCGCTC3' | 5'TAAACCCCATAGTGCTTGCC3' | 175 | 30 | 94℃-40 seconds; 55℃-30 seconds; 72℃-30 seconds |  |
| RXRγ | [NM_009107](http://www.ncbi.nlm.nih.gov/entrez/viewer.fcgi?db=nucleotide&val=42476335) | 5'TTCTTCAAAAGGACCATCAGG3' | 5'CGTTCATGTCACCGTAGGATTCT3' | 289 | 30 | 94℃-40 seconds; 57℃-30 seconds; 72℃-30 seconds |  |
| CYP26a1 | [NM_007811](http://www.ncbi.nlm.nih.gov/entrez/viewer.fcgi?db=nucleotide&val=178057350) | 5'TTCTGCAGATGAAGCGCAGG3' | 5'TTTCGCTGCTTGTGCGAGGA3' | 211 | 35 | 94℃-40 seconds; 60℃-30 seconds; 72℃-30 seconds |  |
| Raldh2 | [X99273](http://www.ncbi.nlm.nih.gov/entrez/viewer.fcgi?db=nucleotide&val=1430868) | 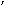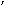5'TTGCAGATGCTGACTTGGAC3' | 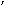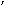5'TCTGAGGACCCTGCTCAGTT3' | 201 | 35 | 94℃-40 seconds; 60℃-30 seconds; 72℃-30 seconds |  |
| Elk1 | [NM_007922](http://www.ncbi.nlm.nih.gov/entrez/viewer.fcgi?db=nucleotide&val=116292181) | 5'TGCTCCCCACACATACCTTGA3' | 5'ACTGGACGGAAACTGGAAGGA3' | 134 | 27 | 94℃-40 seconds; 55℃-30 seconds; 72℃-30 seconds |  |
| GAPDH | [NM_008084](http://www.ncbi.nlm.nih.gov/entrez/viewer.fcgi?db=nuccore&val=126012538) | 5'ACTCACGGCAAATTCAACGG3' | 5'ACGTCAGATCCACGACGGAC3' | 586 | 20 | 94℃-40 seconds; 61℃-30 seconds; 72℃-40 seconds |  |
