# Supplementary material for: All-trans retinoic acid promotes neural lineage entry by pluripotent embryonic stem cells via multiple pathways
Source: BMC Cell Biol. 2009 Jul 30;10:57. doi: 10.1186/1471-2121-10-57 (PMC2728515; doi:10.1186/1471-2121-10-57)
Supplement: Additional file 8 — Q-PCR primer specifications. [file 1471-2121-10-57-S8.doc]

**Table S2: Q-PCR Primer Specifications.**

| **Gene** | **Accession no.** | **Forward primer** | **Reverse primer** |
| --- | --- | --- | --- |
| CYP26a1 | [NM_007811](http://www.ncbi.nlm.nih.gov/entrez/viewer.fcgi?db=nucleotide&val=178057350) | 5'TCTCCAACCTGCACGATTCC3' | 5'CGGCTGAAGGCCTGCAT3' |
| Elk1 | [NM_007922](http://www.ncbi.nlm.nih.gov/entrez/viewer.fcgi?db=nucleotide&val=116292181) | 5'CAGGAATGACAGGCCAAGGT3' | 5'GGCCCGAGCGCATGTAT3' |
| Nestin | [NM_016701](http://www.ncbi.nlm.nih.gov/entrez/viewer.fcgi?db=nucleotide&val=50363231) | 5'GGTCACTGTCGCCGCTACTC3' | 5'CGGACGTGGAGCACTAGAGAA3' |
| Oct3/4 | [NM_013633](http://www.ncbi.nlm.nih.gov/entrez/viewer.fcgi?db=nuccore&val=125490391) | 5'TGGCGTGGAGACTTTGCA3' | 5'GAGGTTCCCTCTGAGTTGCTTTC3' |
| Pax6 | [NM_013627](http://www.ncbi.nlm.nih.gov/entrez/viewer.fcgi?db=nuccore&val=146134521) | 5'CAAACCTGTCTCCTCCTTCACA3' | 5'GGTGAGGGCGGTGTCTGT3' |
| RAR alpha | [NM_009024](http://www.ncbi.nlm.nih.gov/entrez/viewer.fcgi?db=nucleotide&val=116734872) | 5'AGTACTGCCGGCTGCAGAA3' | 5'CGTTTCGCACCGACTCCTT3' |
| RPL19 | [NM_009078](http://www.ncbi.nlm.nih.gov/entrez/viewer.fcgi?db=nuccore&val=6677772) | 5'GACGGAAGGGCAGGCATATG3' | 5'TGTGGATGTGCTCCATGAGG3' |
| Sfrp2 | [NM_009144](http://www.ncbi.nlm.nih.gov/entrez/viewer.fcgi?db=nuccore&val=214010193) | 5'CGTGAAACGGTGGCAGAAG3' | 5'TGCAGCTTGCGGATGCT3' |
